# Role of the Hedgehog Signaling Pathway in Regulating the Behavior of Germline Stem Cells

**DOI:** 10.1155/2017/5714608

**Published:** 2017-08-13

**Authors:** Shiqin Li, Meng Wang, Yanghui Chen, Wei Wang, Junying Wu, Chengpeng Yu, Yuehui Zheng, Zezheng Pan

**Affiliations:** Jiangxi Medical College, Nanchang University, Nanchang, China

## Abstract

Germline stem cells (GSCs) are adult stem cells that are responsible for the production of gametes and include spermatogonial stem cells (SSCs) and ovarian germline stem cells (OGSCs). GSCs are located in a specialized microenvironment in the gonads called the niche. Many recent studies have demonstrated that multiple signals in the niche jointly regulate the proliferation and differentiation of GSCs, which is of significance for reproductive function. Previous studies have demonstrated that the hedgehog (Hh) signaling pathway participates in the proliferation and differentiation of various stem cells, including GSCs in *Drosophila* and male mammals. Furthermore, the discovery of mammalian OGSCs challenged the traditional opinion that the number of primary follicles is fixed in postnatal mammals, which is of significance for the reproductive ability of female mammals and the treatment of diseases related to germ cells. Meanwhile, it still remains to be determined whether the Hh signaling pathway participates in the regulation of the behavior of OGSCs. Herein, we review the current research on the role of the Hh signaling pathway in mediating the behavior of GSCs. In addition, some suggestions for future research are proposed.

## 1. Introduction

Stem cells have many possible applications based on their ability for differentiation. At present, advances in regenerative treatments with stem cells are progressing dramatically, bringing hope for individuals with certain complicated and refractory diseases, such as maculopathy and nerve injury [1, 2]. GSCs are adult stem cells responsible for the production of gametes. The proper maintenance of GSCs is regulated by various signals in the surrounding microenvironment (the niche). In recent years, the hedgehog (Hh) signaling pathway was identified as a relevant regulator of GSCs through its direct regulation of GSCs and indirect regulation of the niche surrounding GSCs to affect GSCs. Because of their ability for differentiation, GSCs have possible applications in reproductive medicine. Here, we review and, respectively, discuss the effect of the Hh signaling pathway on the proliferation and differentiation of SSCs in *Drosophila* and male mammals, the migration of OGSCs from the ovarian surface epithelium (OSE) to the ovary cortex, and the development of OGSCs in the ovary cortex of mammals.

## 2. Hedgehog Signaling Pathway

In 1980, when Nusslein-Volhard and Wieschaus were screening genes that affected the growth of fly larvae, they found that a certain mutated gene caused hedgehog-like spiked protrusions on the ventral side of *Drosophila* embryos; thus, the gene was named *Hh* [3]. Afterwards, three genes, *Shh*, *Dhh*, and *Ihh*, homologous to *Hh*, were found in vertebrates. The proteins encoded by all of the above genes can activate the Hh signaling pathway, which can regulate the function of stem cells, repair damaged cellular tissue, and maintain cellular structure [4, 5].

The classical Hh signaling pathway is composed of the Hh ligand, a membrane protein receptor complex of patched (Ptc) and smoothened (Smo) proteins, nuclear transcription factors, and target genes. *Hh* encodes the Hh precursor protein, which turns into the Hh ligand protein after self-cleavage and palmitoylation. The Hh ligand protein acts on target cells through paracrine or autocrine modes of action with the help of the dispatched (Disp) protein [6, 7]. Interference hedgehog (iHOG; CDO is homologous to it in mammals) and brother of iHOG (BOI; BOC is homologous to it in mammals) are two transmembrane proteins in target cells that can help to reinforce the interaction between Hh and Ptc [8, 9]. In the absence of Hh, Ptc represses the activity of Smo, which inhibits the transduction of Hh signaling via Smo. In contrast, when Hh binds with Ptc, the inhibition of Smo is relieved, which promotes the phosphorylation of the downstream complex SuFu/Ci/Fu (in nonmammals) or SuFu/Gli/Kif7 (in mammals) and releases the nuclear transcription factors Ci or Gli (there are three types of Gli factors in mammals: Gli1, Gli2, and Gli3). Subsequently, transcription factors enter the nucleus to directly regulate the transcription of target genes. In addition, it has been shown that Hh signaling transduction requires the participation of primary cilium.

Generally, nuclear transcription factors are divided into transcription activators (CiA/GliA) and transcription repressors (CiR/GliR) [10]. In mammals, the activities of the proteins Gli1 and Gli2 are similar, and they mainly function as a transcription factor as does GliA. However, Gli3 mostly functions as a transcription repressor as does GliR. In the absence of Hh ligand, Gli3 is restricted to a microtubule complex composed of Kif7 (kinesin family member 7), SuFu (suppressor of fused), CK1 (casein kinase 1), PKA (protein kinase A), and Gsk3*β* (glycogen synthase kinase 3 beta) and is phosphorylated by PKA, GSK3*β*, and CK1. Then, Gli3 is recognized by the E3 ubiquitin ligase *β*TrCP (beta-transducin repeat-containing proteins). Meanwhile, the transcriptional activation region at the C-terminal end is removed, and Gli3 becomes a transcription repressor (GliR), which enters the cell nucleus and inhibits the transcription of target genes. When the Hh ligand is present, activated Smo mediates the phosphorylation of SuFu/Gli/Kif7 complexes that release Gli (mainly Gli1), which blocks the hydrolysis process of the transcriptional activation region at the C-terminal end. Then, the full-length Gli acts like GliA to promote the transcription of target genes. It has been proven that *Hh*, *Gli1*, *Ptc*, and *Bmp* are target genes for the classical Hh signaling pathway.

## 3. Overview of Germline Stem Cells (GSCs)

GSCs are restoring cells in the gonads that have the ability to differentiate into germ cells. They exist in a microenvironment (niche) in the gonads that contain somatic cells. These somatic cells cooperatively regulate the proliferation and differentiation of GSCs. GSCs are usually called spermatogonial stem cells (SSCs) in males [11] and ovarian germline stem cells (OGSCs) in females [12].

### 3.1. Spermatogonial Stem Cells (SSCs)

SSCs in *Drosophila* gonads are located in a niche composed of SSCs, hub cells (HCs), and cyst stem cells (CySCs). In *Drosophila*, at the apex of the bilateral testicle, there is a cluster of HCs. One side of the cluster connects with the basement membrane of the testicle; the other side is the binding site for SSCs and CySCs. Usually, there are six to nine SSCs on one side of the testicle, and each SSC is isolated by two irregular-shaped CySCs. During spermatogenesis, SSCs divide asymmetrically into two daughter cells. One cell adheres to HCs to maintain self-renewal, while the other separates from the HCs and forms a gonialblast (GB), which enters the differentiation stage. CySCs divide into two cyst cells, which surround the GB and regulate its proliferation and differentiation [13–15].

In 1994, SSCs were first discovered in mammals in the basement membrane of the mouse testis seminiferous tubule, and they account for 0.02%–0.03% of the total cells in the testis [16]. They are located in a niche that is mainly composed of SSCs, Sertoli cells (SCs), Leydig cells (LCs), peritubular myoid (PTM) cells, and other spermatogonia and spermatic cells in various differentiation phases. Spermatogonia in the testes are classified into three types, including type A, type intermediate, and type B. Type A can be subdivided into type A single (As), type A paired (Apr), and type A aligned (Aal). Among them, type As has the lowest differentiation degree and is currently widely recognized as SSCs [11, 17].

### 3.2. Ovarian Germline Stem Cells (OGSCs)

OGSCs in *Drosophila* are located in a niche composed of cap cells (CpCs), terminal filament cells (TFCs), and escort cells (ECs). The niche is located in the apex of the oviduct in the bilateral germarium. In general, there are two or three OGSCs in each germarium. They adhere to CpCs through the E-calcium adhesion protein. OGSCs can asymmetrically divide into two daughter cells: one cell turns into a new OGSC and continues to adhere to CpCs while the other one separates itself from CpCs and proceeds to becoming an oocyte latterly [18, 19]. Furthermore, Zou et al. [20] first segregated a type of cell from the postnatal mouse ovarian surface epithelium (OSE) in 2009. These isolated cells could coexpress the germ cell-specific marker Mvh and the stem cell markers OCT-4 and SSEA-1 [21, 22], and they are able to differentiate into oocyte-like cells in vitro. This type of cell is known as an OGSC. Then, in 2012, White et al. [23] further isolated mitotically active germ cells from the ovaries of reproductive-age women that could generate oocytes in vitro and in vivo. Afterwards, in 2014, Dunlop et al. [24] purified OGSCs from the adult and bovine ovarian cortex. Hence, OGSCs in mammals especially in humans probably have great significance for the reproductive ability of female mammals and treatment of diseases related to germ cells. However, OGSCs are present in small amounts in the ovary. Thus, further studies on the molecular regulatory mechanism of OGSC behavior are especially crucial.

## 4. Effect of the Hedgehog Signaling Pathway on SSCs

### 4.1. Effect of the Hedgehog Signaling Pathway on *Drosophila* SSCs

In *Drosophila*, the hedgehog signaling pathway regulates the proliferation and differentiation of SSCs via an indirect mechanism. Although SSCs are closely conglutinated with HCs, SSCs cannot express Ptc and Smo and fail to receive Hh signaling from HCs. Furthermore, the Hh ligand originating from HCs directly binds with Ptc in CySCs, which results in the upregulation of the transcription of target genes, including *Hh* and *Bmp* (bone morphogenetic protein) [15]. The increased expression of Hh participates in the positive feedback of Hh signaling pathway activity in CySCs. Moreover, BMPs specifically activate the BMP signaling pathway in SSCs, which inhibits the transcription of *bag-of-marbles* (*Bam*), which encodes Bam (differentiation-associated factor), to sustain the undifferentiated status of SSCs [25]. Hence, Hh signaling in the niche is an indirect signal that helps to sustain the proper number of SSCs. An overactivated or insufficiently activated Hh signaling pathway in CySCs would result in a decrease in SSCs. When the Hh signaling pathway is overactivated in CySCs, the number of SSCs sharply decreases probably because of the competition of abnormal proliferous CySCs. However, when the Hh signaling pathway is insufficiently activated, the number of CySCs decreases, and they fail to form the proper BMP concentration in the niche, which negatively affects the regeneration of SSCs [26]. Hence, controlling Hh signaling accurately in the SSC niche helps maintain SSCs, and it likely benefits the *Drosophila* spermatogenic cycle.

### 4.2. Hedgehog Signaling Pathway Regulates the Proliferation and Differentiation of Mammalian SSCs

In mammals, the Hh signaling pathway regulates the proliferation and differentiation of SSCs in direct and indirect ways. Previous studies mainly considered that Hh signaling could indirectly affect the cellular behavior of SSCs through somatic cells in the niche. However, recent research revealed that SSCs could secrete Hh ligand and directly activate Hh signaling pathway in SSCs.

#### 4.2.1. Hedgehog Signaling Pathway Indirectly Regulates the Proliferation and Differentiation of Mammalian SSCs

**Figure 1:**
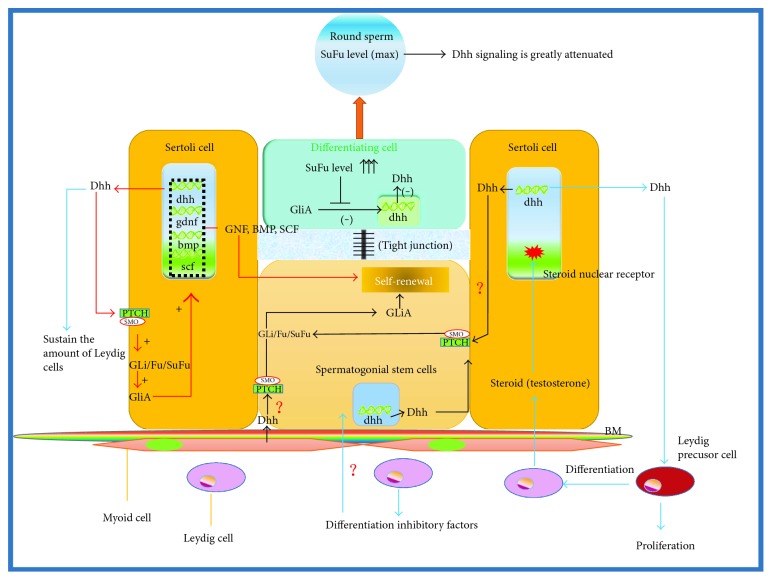
Effect of the Hh signaling pathway on the regulation of proliferation and differentiation of SSCs in mammals. (Arrowhead in red in this figure) Hh secreted by SCs can promote the expression of various factors in SCs, which helps to maintain SSC proliferation. (Arrowhead in blue in this figure) Dhh produced by SC is crucial for LCs for maintaining the number of LCs and expression of testosterone, which is favorable for the differentiation of SSCs. LCs also produce some undefined inhibitory factors that inhibit SSC differentiation. (Arrowhead in black in this figure) as SSCs differentiate, the expression of SuFu (a negative regulator of the Hh pathway) constantly increases. It inhibits the activity of the Hh pathway, which is beneficial for the differentiation of SSCs (arrowhead in black in this figure).

In mice, SSCs directly adhere to adult SCs in the niche. During the adult SC period, the transcription and sustained expression of Dhh can be detected in SCs. After secreting Dhh, adult SCs accept Hh signaling and promote the expression of glial cell-derived neurotrophic factor (GDNF), bone morphogenetic protein (BMP), and stem cell factor (SCF). These proteins can further activate other signaling pathways in SSCs, such as BMP, PI3K-Akt, Src, and RAS/ERK1/2, which inhibit the differentiation of SSCs and sustain a certain amount of SSCs [27–29] (Figure 1, arrowhead in red).

LCs and their precursor stem cells are located beneath the basement membrane of seminiferous tubules, and Ptc and Smo are on the surface of precursor stem cells. Dhh secreted by SCs affects the differentiation of Leydig lineage cells and establishment of the mature LC system, which is of great significance to the growth and development of SSCs [30, 31]. First, Dhh secreted by SCs is essential for the secretion of steroid hormones such as testosterone. Testosterone is indispensable in the process of spermatogenesis as it promotes the differentiation of SSCs into spermatids. Moreover, there are testosterone receptors in SCs. The hyposecretion of testosterone results in developmental defects in SCs, which negatively affect SSC differentiation [32]. Second, Dhh promotes the differentiation of LC precursor stem cells into adult LCs, and it is worth emphasizing that activated Hh signaling is a prerequisite for this differentiation process [33]. Third, research shows that there is primary cilium in poorly differentiated LCs in human adult testes [34]. However, the cilium will disappear when LCs develop into mature cells, which probably explains why LC precursor stem cells are sensitive to Hh signaling. Moreover, it also illustrates that Dhh secreted by SCs is important in establishing a mature LC system, which is ultimately favorable to SSC differentiation [35]. In addition, it has been reported that some undefined products seem to contribute to the inhibition of spermatogonial differentiation in irradiated rats [33]. Hence, during the process of SSC proliferation and differentiation, the balance between SCs and LCs regulated by Hh signaling is crucial [36] (Figure 1, arrowhead in blue).

#### 4.2.2. Hedgehog Signaling Pathway Directly Regulates the Proliferation and Differentiation of Mammalian SSCs

In 2014, Sahin et al. [37] found that the transcription of the Hh pathway components *Hh*, *Ptc*, *Smo*, and *Gli1* could be detected in an environment that solely contains undifferentiated SSCs. Moreover, SSCs did not differentiate and the number of SSCs increased in the first several months. Thus, they proposed that SSCs could maintain self-renewal before differentiation through an autocrine loop of Hh signaling. Meanwhile, SuFu is a negative regulatory factor in the Hh pathway, and it cannot be detected in earlier differentiation periods of SSCs. However, when SSCs develop into mature spermatids, the expression of SuFu constantly increases and inhibits Gli transcriptional activity, which results in the suppression of Dhh signaling in advanced circular sperm cells [38–40]. Therefore, as SSCs differentiate, their Hh signaling activity continuously decreases. The reason why SuFu is not expressed in earlier periods remains unknown. In addition, SSCs are the direct target cells of Hh ligand, which suggests that Dhh secreted by SCs perhaps could directly regulate the proliferation of SSCs. However, the regulatory effect of Dhh produced by SCs or PMCs on SSCs needs to be explored further (Figure 1, arrowhead in black).

## 5. Effect of the Hedgehog Signaling Pathway on OGSCs

### 5.1. Effect of the Hedgehog Signaling Pathway on *Drosophila* OGSCs

**Figure 2:**
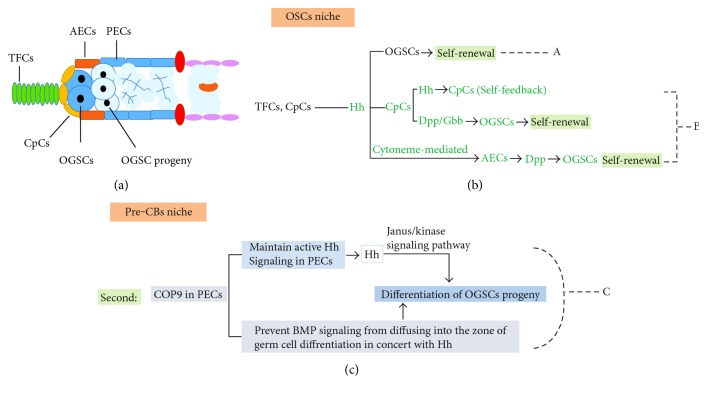
Effect of the Hh signaling pathway on the regulation of proliferation and differentiation of OGSCs in *Drosophila*. (a) OGSCs and their progeny are located in a niche that is composed of various somatic cells. (b) (A) Hh secreted from TFCs and CpCs can promote the self-renewal of OGSCs directly. (B) Hh originating from TFCs and CpCs can promote CpCs and AECs to secrete Gbb or Dpp, which is indirectly beneficial for the self-renewal of OGSCs. (c) (C) COP9 is important for maintaining the activity of the Hh pathway in PECs. The COP and Hh axis helps to prevent Dpp from diffusing. Moreover, COP9 can downregulate Dpp expression in PECs. A low concentration of Dpp antagonizes OGSC progeny differentiation.

Hh signaling in the niche of OGSCs can suppress the differentiation of OGSCs in direct and indirect ways. Firstly, Hh signaling has been proposed to directly signal OGSCs and control the maintenance of OGSCs (Figure 2(b), A). Secondly, TFCs and CpCs are referred to as apical cells and can secret Hh. The coreceptor of Hh iHOG/BOI can bind Hh with high affinity and sequester Hh on the surface, which inhibits Hh diffusion and forms a positive feedback of Hh pathway in apical cell. As target genes of Hh pathway, the expression of Hh increased [41, 42]. Then, CpCs deliver Hh signaling to AECs (ECs located in the anterior part of the germarium) and promote the transcription of the target genes *Decapentaplegic* (*Dpp*) and *glass bottom boat* (*Gbb*) (both belong to the *Bmp* family), in AECs [27, 43]. Hh secreted by CpCs also directly enhances the transcription of *Dpp* in CpCs. Eventually, BMP (Dpp) signaling in the OGSCs niche is intensified. As a result, Dpp suppresses the expression of *Bam* and inhibits the differentiation of OGSCs. As OGSCs differentiate, the expression of Fused (Fu), a positive regulator in the Hh pathway, is constantly increased. Moreover, Fu mediates the ubiquitination and proteolysis of thickveins (Tkv, a BMP receptor) in OGSCs, which is beneficial for the differentiation of OGSCs [19, 44–47] (Figure 2(b), B).

When OGSC progeny separates from CpCs, PEC- (ECs located in the posterior part of the germarium-) derived but not CpC-derived Hh starts to participate in the differentiation process. It was discovered that maintaining the activation of the Hh signaling pathway and secretion of Hh ligand in PECs requires the presence of COP9 (also known as the CSN complex) in ECs [48]. Moreover, the COP9-Hh axis in PECs can partly prevent the diffusing of Dpp and promote the differentiation of OGSC progeny. In addition, JAK/STAT signaling promotes Dpp expression, whereas Hh signaling from PECs suppresses Dpp expression by antagonizing JAK/STAT signaling, which favors the differentiation of OGSC progeny. Therefore, it seems that Hh protein in the niche promotes the differentiation of OGSC progeny via suppressing the transcription of *Dpp* in a non-Hh signaling mechanism [49] (Figure 2(c), C).

### 5.2. Effect of the Hedgehog Signaling Pathway on Mammalian OGSCs

OGSCs in mammals are located in a single layer of epithelial cells with tight junctions in the OSE. In almost all species, it is not possible to observe obvious OGSC division in the normal OSE, and most research concerning OGSC function involves OGSC transplantation experiments. Thus, it is more reasonable to discuss the effect of Hh signaling pathway on OGSCs in normal OSE or in the ovary cortex after transplantation [50–52]. Moreover, it will be interesting to determine whether artificially altering the activity of Hh pathway will benefit ovum regeneration.

#### 5.2.1. Effect of the Hedgehog Signaling Pathway on the Migration of OGSCs in the OSE

**Figure 3:**
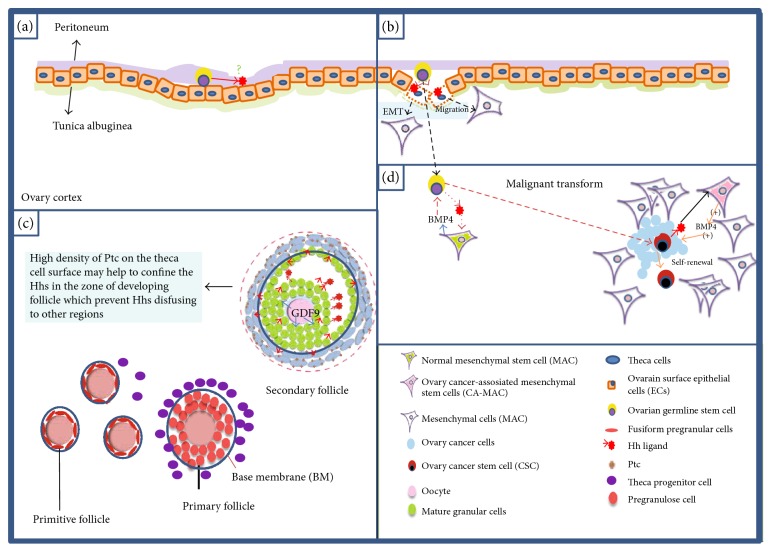
Effect of the Hh signaling pathway on the behavior of OGSCs in mammals. (a) It is not clear whether OGSCs in the ovarian surface epithelium (OSE) secrete Hh. (b) It is thought that the EMT could help OGSCs migrate to the ovary cortex from the OSE. (c) Ptc in TCs assists in preventing the Hh ligand from diffusing to other developing follicles and the interstitial stroma. (d) CSCs of ovarian cancer are likely to be the malignant transformation products of OGSCs. There is a BMP4-Hh feedback loop between CSCs and CA-MACs that promotes CSC proliferation and maintains the CSC number. It has not been determined whether there is a similar BMP4-Hh feedback loop between OGSCs and MSCs.

We proposed a hypothesis that the special structure of the OSE restricts the activity of OGSCs, but Hh signaling could help OGSCs migrate into the ovary cortex. Under natural conditions, the outside of the OSE is covered by the peritoneum, and the underside of the OSE adjoins the ovary cortex [53–55]. However, there is a layer of dense-constructed ovary tunica albuginea between the OSE and the ovary cortex. Restricted by this physiological structure, OGSCs seem to be isolated in an area that is distant from the ovary cortex, which contains various somatic cells such as granulosa cells (GCs), theca cells (TCs), and mesenchymal cells (MCs). Thus, in the normal ovary, it is difficult to predict how Hh signaling regulates the proliferation, differentiation, or any other cellular behavior of OGSCs through other somatic cells. Epithelial cells in the OSE are equipped with both epithelial and mesenchymal phenotypes. They can undergo epithelial-mesenchymal transition (EMT), which changes the tight junctions between epithelial cells into looser junctions between mesenchymal cells. Previous studies revealed that Hh signaling pathway participates in EMT in other tissues [56–58]. However, Gli1 transcription is not observed in the OSE, seemingly indicating that Hh pathway is not activated in the OSE in the normal state. However, the Hh signaling pathway participates in the migration of epithelial ovarian cancer cells into the ovary cortex. This suggests that the Hh pathway is possibly related to the migration of OGSCs into the ovary cortex. Once OGSCs shift to the cortex, they enter into an entirely different environment that contains various biosignals that probably promote the proliferation or differentiation of OGSCs (Figure 3(b)).

#### 5.2.2. Effect of the Hedgehog Signaling Pathway on the Proliferation and Differentiation of OGSCs

It is not clear whether somatic cells in the ovary cortex regulate the function of OGSCs through the Hh signaling pathway. During the initial forming period of the mammalian fetal ovary, the transcription levels of *Hh*, *Ptc*, and *Gli* are very low [59]. After birth, the primordial oocyte of the primary follicle secretes the differentiation factor GDF9 (growth differentiation factor 9) [60], inducing granulosa cells to secrete Dhh and Ihh. Then, Dhh and Ihh continue to induce progenitor TCs to differentiate into TCs to promote follicle development [61–64]. In the ovary, GCs are the main source of Hh protein, while Ptc is the most abundant on the surface of TCs. Ptc not only binds with Hh but also works in preventing Hh signaling from diffusing [63, 65]. Hence, Ptc in TCs distributed around the outer sphere of the follicle seems to help prevent Hh from diffusing into nearby follicles or interstitial cells. Thus, when we injected OGSCs into the ovary cortex, they came into direct contact with the somatic cells (Figure 3(c)). In 2015, a study reported that cancer stem cells of ovary cancer are very likely a malignant transformed product of OGSCs [66]. Furthermore, a previous study found that there is a BMP4-Hh-positive feedback loop between CSCs (cancer stem cells) of ovary cancer and CA-MACs (cancer-associated mesenchymal stem cells), which enhances the proliferation of CSCs. Meanwhile, CSCs produce Hh to activate the BMP signaling pathway in CA-MSCs [67]. Then, the overexpression of BMP4 suppresses CSCs to differentiate, resulting in the overexpression of Hh. Although CSCs can secret Hh, it is not clear whether CSCs have the ability to secrete Hh before or after canceration. Until now, no study has mentioned whether OGSCs can produce Hh. Normal MSCs (mesenchymal stem cells) can also produce BMPs but at lower levels compared with CA-MACs, so it is unclear whether OGSCs in the normal ovarian stroma together with MACs form a positive feedback loop like the BMP4-Hh feedback loop between CSCs and CA-MACs and promote the proliferation of OGSCs (Figures 3(a) and 3(d)). Furthermore, Park et al. [68] found that BMP4 could promote the differentiation of OGSCs into oocytes via Smad1/5/8 signaling in mice. This result was counter to the assumption that BMP4 helps to sustain the self-renewal of OGSCs. However, the result may depend on the dosage of BMP. Therefore, we conclude that the proliferation or differentiation of OGSCs varies with different concentrations of BMP4 conducted by Hh signaling from OGSCs. This resembles the dual regulation in *Drosophila* when Hh signaling stimulates other cells to secrete BMP protein.

Can somatic cells in the ovary regulate OGSC development as in *Drosophila*? Grieve et al. [51] found that stage-specific granulosa cells could induce the expression of oocyte-specific genes in embryonic stem cells under coculture conditions. He proposed that somatic cells are important for facilitating the differentiation of stem cells into functional oocytes. GCs can secrete the Hh ligand, but it is not clear whether the Hh pathway is involved in the proliferation and differentiation of OGSCs under coculture with OGSCs. However, it would perhaps be more convincing if we use OGSCs to replace the oogonia in primary follicles and then observe follicle development. A developing follicle is a good model for studying the relationship between GCs, Hh, and OGSCs. However, it is not clear whether Ptc and Smo are in OGSCs, which if so would indicate that altering Hh pathway activity would regulate the proliferation of OGSCs.

## 6. Perspective

According to the current research progress in this area, the following questions need to be thoroughly addressed in the near future.

Currently, many studies have indicated that the Hh signaling pathway has a strong effect on the proliferation and differentiation of SSCs. What we are mostly interested is whether we can artificially alter the self-renewal or differentiation process via regulating Hh signaling in the SSC niche, thereby improving the reproductive ability of animals. For example, some animals with azoospermia cannot produce a sufficient number of sperm and lose their reproductive ability because of the dysfunction of SSCs or the low number of SSCs. A sufficient number of SSCs is a prerequisite for a normal spermatogenic ability in males. In 2014, it was determined in testicular biopsies that the ectopic expression of Shh results in the absence of spermatocytes and increased numbers of LCs in the testes [69]. We discussed in this article that LCs have dual roles in regulating SSC physiology. Hence, the proper regulation of Hh signaling in the niche is vital to maintain the number of SSCs. In addition, apart from proliferation and differentiation, apoptosis is also an important physiological process of SSCs that could impact the number of SSCs. Moreover, the Hh signaling pathway possibly takes part in antiapoptosis, but there has been no relevant study. In *Drosophila*, the JAK-STAT signaling pathway, whose functions overlap with those of the Hh signaling pathway, enhances the proliferation of SSCs and the overexpression of the antiapoptosis protein DIAPI in cells, which helps sustain the activity of SSCs [70]. Thus, the Hh pathway probably functions in the antiapoptosis process. When SSCs differentiate, the increase in the SuFu level inhibits the activity of the Hh pathway. While there is little SuFu in SSCs, it is not known whether any substance other than SuFu antagonizes ectopic Hh signaling to maintain the proper number of SSCs. In short, regardless of what promotes the proliferation, differentiation, or antiapoptosis of SSCs via altering Hh signaling pathway activity, these results all provide some new ideas for clinical therapeutic methods for treating azoospermia or other diseases related to SSCs.

OGSCs are considered one of the possible cells in animals that can generate oocytes. Regulating OGSC function can be used to help those who wish to lengthen their reproductive life span or to treat animals or humans with germ cell dysfunction to restore their reproductive ability. Whether changing the Hh signaling pathway in OGSCs can affect the formation of primordial follicles in mammals and whether any effects on the proliferation and differentiation of OGSCs are caused by Hh signaling regulation still require further investigation. Previous studies have shown that Hh signaling can influence the development of TCs and hinder ovulation in mammals [71], but there has been no report on the effect of Hh signaling on OGSCs and primordial follicle formation. However, other signaling pathways, such as Hippo-YAP, Notch, and WNT [72–74], closely related to the Hh signaling pathway, have been found to be involved in regulating the proliferation and differentiation of OGSCs, as well as influencing the formation and development of mammalian primordial follicles. For example, Ci [75], a component of the Hh pathway, suppresses the activity of the Hippo signaling pathway kinase cascade in *Drosophila* ovary somatic cells, which eventually promotes the differentiation of OGSCs. Meanwhile, the Hippo-YAP signaling pathway has a negative regulatory effect on the generation and development of mammalian follicles [76], which probably indicates that the activation of the Hh pathway can enhance the growth and development of mammalian follicles via regulating the behavior of OGSCs.
